# *catena*-Poly[[aquabis[*N*-(pyridin-3-yl)isonicotinamide-κ*N* ^1^]copper(II)]-μ-fumarato-κ^2^ *O* ^1^:*O* ^4^]

**DOI:** 10.1107/S1600536812047101

**Published:** 2012-11-24

**Authors:** Sultan H. Qiblawi, Robert L. LaDuca

**Affiliations:** a Lyman Briggs College, Department of Chemistry, Michigan State University, East Lansing, MI 48825 USA

## Abstract

In the title compound, [Cu(C_4_H_2_O_4_)(C_11_H_9_N_3_O)_2_(H_2_O)]_*n*_, Cu^II^ ions on crystallographic twofold rotation axes are coordinated in a square pyramidal environment by two *trans* O atoms belonging to two monodentate fumarate anions, two *trans* isonicotinamide pyridyl N-donor atoms from monodentate, pendant 3-pyridyl­isonicotinamide (3-pina) ligands, and one apical aqua ligand, also sited on the crystallographic twofold rotation axis. The exobidentate fumarate ligands form [Cu(fumar­ate)(3-pina)_2_(H_2_O)]_*n*_ coordination polymer chains that are arranged parallel to [001]. In the crystal, these polymeric chains are anchored into supra­molecular layers parallel to (100) by O—H⋯O hydrogen bonds between aqua ligands and unligating fumarate O atoms, and N—H⋯O(=C) hydrogen bonds between 3-pina ligands. In turn, the layers aggregate by weak C—H⋯N and C—H⋯O hydrogen bonds, affording a three-dimensional network.

## Related literature

For the preparation of 3-pyridyl­isonicotinamide, see: Gardner *et al.* (1954 ▶). For the preparation of other dicarboxyl­ate coordination polymers containing 3-pyridyl­isonicotinamide, see: Kumar (2009 ▶).
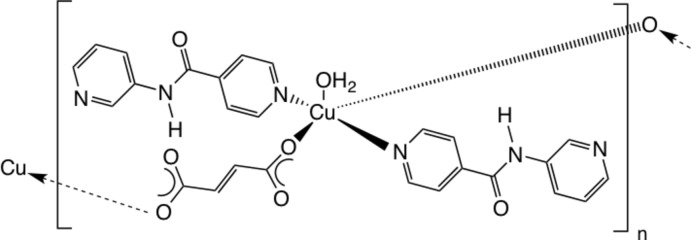


## Experimental

### 

#### Crystal data

[Cu(C_4_H_2_O_4_)(C_11_H_9_N_3_O)_2_(H_2_O)]
*M* *_r_* = 594.04
Monoclinic, 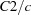

*a* = 29.854 (4) Å
*b* = 5.3535 (7) Å
*c* = 17.353 (2) Å
β = 118.686 (2)°
*V* = 2433.0 (6) Å^3^
*Z* = 4
Mo *K*α radiation
μ = 0.96 mm^−1^
*T* = 173 K
0.25 × 0.13 × 0.09 mm

#### Data collection

Bruker APEXII CCD diffractometer
Absorption correction: multi-scan (*SADABS*; Sheldrick, 1996 ▶) *T*_min_ = 0.794, *T*_max_ = 0.919
9406 measured reflections
2240 independent reflections
1758 reflections with *I* > 2σ(*I*)
*R*_int_ = 0.053

#### Refinement

*R*[*F* ^2^ > 2σ(*F* ^2^)] = 0.036
*wR*(*F* ^2^) = 0.083
*S* = 1.04
2240 reflections
188 parameters
5 restraints
H atoms treated by a mixture of independent and constrained refinement
Δρ_max_ = 0.34 e Å^−3^
Δρ_min_ = −0.36 e Å^−3^

### 

Data collection: *APEX2* (Bruker, 2006 ▶); cell refinement: *SAINT* (Bruker, 2006 ▶); data reduction: *SAINT*; program(s) used to solve structure: *SHELXS97* (Sheldrick, 2008 ▶); program(s) used to refine structure: *SHELXL97* (Sheldrick, 2008 ▶); molecular graphics: *Crystal Maker* (Palmer, 2007 ▶); software used to prepare material for publication: *SHELXL97*.

## Supplementary Material

Crystal structure: contains datablock(s) I, global. DOI: 10.1107/S1600536812047101/lh5556sup1.cif

Structure factors: contains datablock(s) I. DOI: 10.1107/S1600536812047101/lh5556Isup2.hkl

Additional supplementary materials:  crystallographic information; 3D view; checkCIF report

## Figures and Tables

**Table 1:** Hydrogen-bond geometry (Å, °)

*D*—H⋯*A*	*D*—H	H⋯*A*	*D*⋯*A*	*D*—H⋯*A*
O3—H3*O*⋯O1^i^	0.84 (1)	1.83 (1)	2.660 (2)	169 (3)
N2—H2*N*⋯O2^i^	0.88 (2)	2.33 (2)	3.153 (3)	155 (3)
C2—H2⋯O4^ii^	0.95	2.48	3.360 (4)	153
C7—H7⋯O1^iii^	0.95	2.48	3.430 (4)	178
C9—H9⋯N1^iv^	0.95	2.39	3.263 (4)	153
C12—H12⋯O1^v^	0.95	2.43	3.374 (4)	171

Symmetry codes: (i) 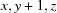; (ii) 
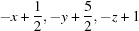
; (iii) 
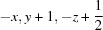
; (iv) 
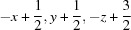
; (v) 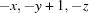.

**Fig. 1.**
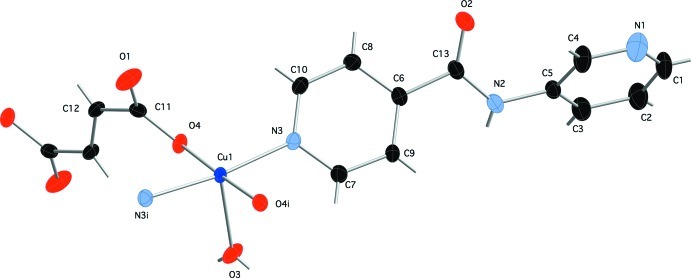
The coordination environment of the title compound, showing 50% probability ellipsoids and atom numbering scheme. Hydrogen atom positions are shown as grey sticks. Color codes: dark blue Cu, red O, light blue N, black C, pink H. Symmetry code: (i) -x, y, -z + 1/2

**Fig. 2.**
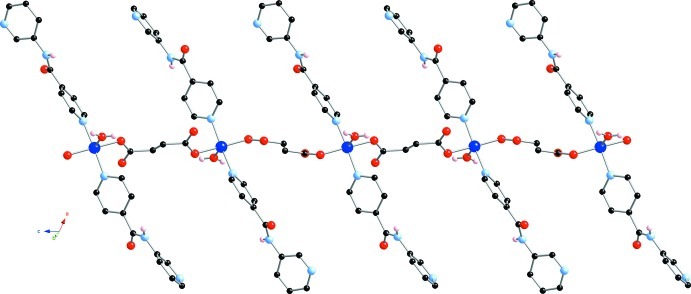
A single [Cu(fumarate)(3-pina)2(H2O)]n coordination polymer chain.

**Fig. 3.**
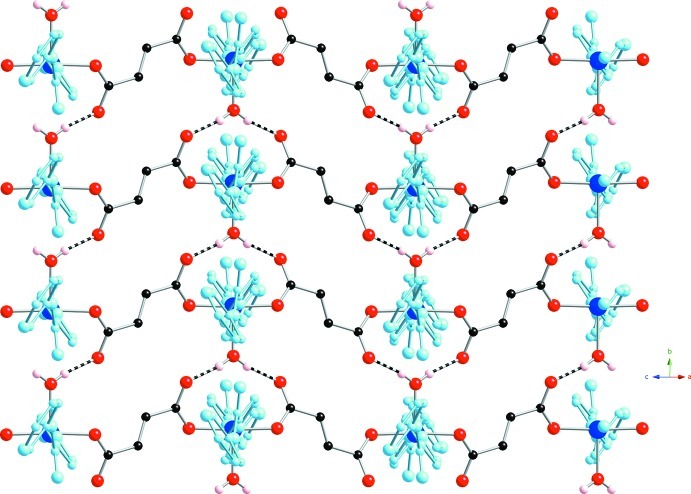
Supramolecular layer of [Cu(fumarate)(3-pina)2(H2O)]n coordination polymer chains. O—H···O hydrogen bonding is shown as dashed lines.

**Fig. 4.**
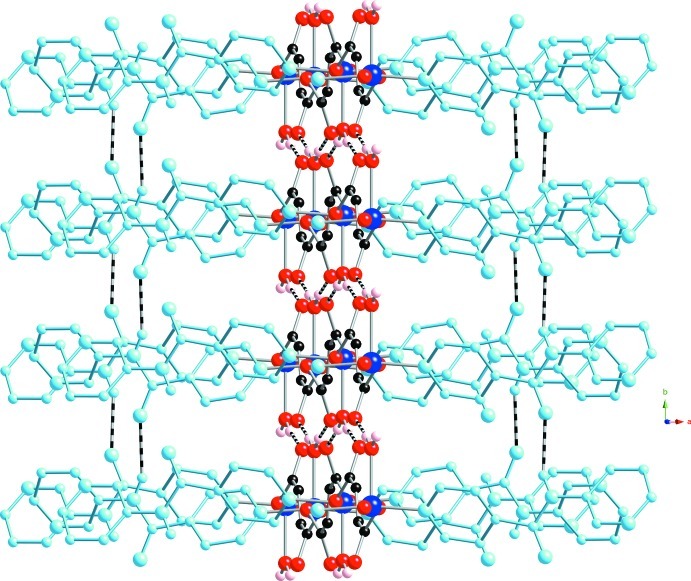
Side view of the supramolecular layer of [Cu(fumarate)(3-pina)2(H2O)]n coordination polymer chains. N—H···O hydrogen bonding between 3-pina amide groups is shown as dashed lines.

**Fig. 5.**
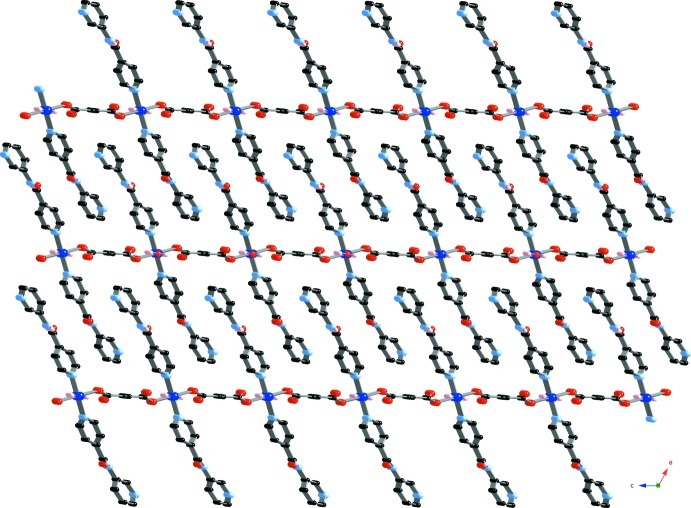
Stacking of supramolecular layers within the title compound.

## supplementary crystallographic information

### Comment

In comparison to divalent metal coordination polymers containing rigid rod dipyridine ligands such as 4,4'-bipyridine, related materials containing the kinked dipodal ligand 3-pyridylisonicotinamide (3-pina) are much less common (Kumar, 2009). The title compound was obtained as blue crystals through the hydrothermal reaction of copper nitrate, fumaric acid, and 3-pina.

The asymmetric unit of the title compound contains a divalent copper atom and an aqua ligand on a crystallographic twofold rotation axis, a 3-pina ligand, and one half of a fumarate ligand whose centroid rests on a crystallographic inversion centre. The copper atom is square pyramidally coordinated (Fig. 1), with the basal plane containing *trans* isonicotinamide pyridyl N atom donors from two 3-pina ligands and *trans* O atom donors from monodentate carboxylate groups belonging to two fumarate ligands. The aqua ligand is located in the apical position.

The Cu atoms are linked by exobidentate, bis(monodentate) fumarate ligands to form [Cu(fumarate)(3-pina)_2_(H_2_O)]_n_ coordination polymer chains that are oriented parallel to [0 0 1] (Fig. 2). Each individual chain is anchored to two others *via* O—H···O pairwise hydrogen bonding (Table 1) between aqua ligands and unligated fumarate O atoms, thereby constructing supramolecular two-dimensional layers arranged parallel to the *bc* crystal planes (Fig. 3). The stability of the layer motifs is enhanced by N—H···O hydrogen bonding between amide groups of adjacent 3-pina ligands (Fig. 4). In turn the layers stack along [1 0 0] in an *AAA* pattern *via* C—H···N interactions mediated by unligated 3-pyridyl N atoms belonging to the pendant 3-pina ligands (Fig. 5), thus forming the three-dimensional structure of the title compound which is also stabilized by weak C—H···O interactions.

### Experimental

Copper(II) nitrate hydrate and fumaric acid were obtained commercially. 3-Pyridylisonicotinamide (3-pina) was prepared *via* a published procedure (Gardner *et al.*, 1954). A mixture of copper nitrate hydrate (86 mg, 0.37 mmol), fumaric acid (42 mg, 0.36 mmol), 3-pina (74 mg, 0.37 mmol) and 10.0 g water (550 mmol) was placed into a 23 ml Teflon-lined Parr acid digestion bomb, which was then heated under autogenous pressure at 393 K for 24 h. Blue needles of the title compound were obtained.

### Refinement

All H atoms bound to C atoms were placed in calculated positions, with C—H = 0.95 Å, and refined in riding mode with *U*_iso_ = 1.2*U*_eq_(C). The H atom within the amide group of the 3-pina ligand was found in a difference Fourier map, restrained with N—H = 0.90 (2) Å and refined with *U*_iso_ = 1.2*U*_eq_(N). The H atoms within the aqua ligand were found in a difference Fourier map, restrained with O—H = 0.85 (2) Å and refined with *U*_iso_ = 1.2*U*_eq_(O).

### Crystal data

**Table:**

[Cu(C_4_H_2_O_4_)(C_11_H_9_N_3_O)_2_(H_2_O)]	*F*(000) = 1220
*M**_r_* = 594.04	*D*_x_ = 1.622 Mg m^−^^3^
Monoclinic, *C*2/*c*	Mo *K*α radiation, λ = 0.71073 Å
Hall symbol: -C 2yc	Cell parameters from 3329 reflections
*a* = 29.854 (4) Å	θ = 2.7–25.3°
*b* = 5.3535 (7) Å	µ = 0.96 mm^−^^1^
*c* = 17.353 (2) Å	*T* = 173 K
β = 118.686 (2)°	Needle, blue
*V* = 2433.0 (6) Å^3^	0.25 × 0.13 × 0.09 mm
*Z* = 4	

### Data collection

**Table:**

Bruker APEXII CCD diffractometer	2240 independent reflections
Radiation source: fine-focus sealed tube	1758 reflections with *I* > 2σ(*I*)
Graphite monochromator	*R*_int_ = 0.053
ω–φ scans	θ_max_ = 25.4°, θ_min_ = 2.4°
Absorption correction: multi-scan (*SADABS*; Sheldrick, 1996)	*h* = −35→35
*T*_min_ = 0.794, *T*_max_ = 0.919	*k* = −6→6
9406 measured reflections	*l* = −20→20

### Refinement

**Table:**

Refinement on *F*^2^	Primary atom site location: structure-invariant direct methods
Least-squares matrix: full	Secondary atom site location: difference Fourier map
*R*[*F*^2^ > 2σ(*F*^2^)] = 0.036	Hydrogen site location: inferred from neighbouring sites
*wR*(*F*^2^) = 0.083	H atoms treated by a mixture of independent and constrained refinement
*S* = 1.04	*w* = 1/[σ^2^(*F*_o_^2^) + (0.0303*P*)^2^ + 4.2344*P*] where *P* = (*F*_o_^2^ + 2*F*_c_^2^)/3
2240 reflections	(Δ/σ)_max_ < 0.001
188 parameters	Δρ_max_ = 0.34 e Å^−^^3^
5 restraints	Δρ_min_ = −0.36 e Å^−^^3^

### Special details

**Table:**

Geometry. All e.s.d.'s (except the e.s.d. in the dihedral angle between two l.s. planes) are estimated using the full covariance matrix. The cell e.s.d.'s are taken into account individually in the estimation of e.s.d.'s in distances, angles and torsion angles; correlations between e.s.d.'s in cell parameters are only used when they are defined by crystal symmetry. An approximate (isotropic) treatment of cell e.s.d.'s is used for estimating e.s.d.'s involving l.s. planes.
Refinement. Refinement of *F*^2^ against ALL reflections. The weighted *R*-factor *wR* and goodness of fit *S* are based on *F*^2^, conventional *R*-factors *R* are based on *F*, with *F* set to zero for negative *F*^2^. The threshold expression of *F*^2^ > σ(*F*^2^) is used only for calculating *R*-factors(gt) *etc*. and is not relevant to the choice of reflections for refinement. *R*-factors based on *F*^2^ are statistically about twice as large as those based on *F*, and *R*- factors based on ALL data will be even larger.

### Fractional atomic coordinates and isotropic or equivalent isotropic displacement parameters (Å^2^)

**Table:**

	*x*	*y*	*z*	*U*_iso_*/*U*_eq_	
Cu1	0.0000	0.98462 (9)	0.2500	0.01332 (15)	
O1	−0.01260 (9)	0.5988 (4)	0.10232 (13)	0.0329 (6)	
O2	0.23784 (7)	0.6328 (4)	0.58520 (13)	0.0277 (5)	
O3	0.0000	1.3863 (5)	0.2500	0.0288 (7)	
H3O	−0.0035 (12)	1.471 (4)	0.2067 (12)	0.035*	
O4	0.01923 (7)	0.9749 (3)	0.15746 (11)	0.0170 (4)	
N1	0.34390 (10)	0.8621 (5)	0.83508 (17)	0.0384 (7)	
N2	0.24546 (9)	1.0496 (5)	0.61401 (16)	0.0232 (6)	
H2N	0.2343 (11)	1.199 (4)	0.5910 (18)	0.028*	
N3	0.07405 (8)	0.9573 (4)	0.34350 (14)	0.0159 (5)	
C1	0.37744 (12)	1.0459 (6)	0.8530 (2)	0.0344 (8)	
H1	0.4076	1.0448	0.9083	0.041*	
C2	0.37059 (12)	1.2372 (6)	0.7956 (2)	0.0339 (8)	
H2	0.3955	1.3652	0.8108	0.041*	
C3	0.32648 (12)	1.2391 (6)	0.7149 (2)	0.0295 (8)	
H3	0.3203	1.3698	0.6739	0.035*	
C4	0.30186 (11)	0.8643 (6)	0.75726 (19)	0.0292 (7)	
H4	0.2777	0.7335	0.7438	0.035*	
C5	0.29173 (10)	1.0479 (5)	0.69522 (18)	0.0201 (6)	
C6	0.16993 (10)	0.8946 (5)	0.48796 (18)	0.0187 (6)	
C7	0.09141 (10)	1.1150 (5)	0.41137 (18)	0.0199 (6)	
H7	0.0702	1.2498	0.4094	0.024*	
C8	0.15215 (10)	0.7307 (5)	0.41788 (18)	0.0195 (6)	
H8	0.1725	0.5933	0.4188	0.023*	
C9	0.13897 (10)	1.0906 (5)	0.48445 (18)	0.0214 (7)	
H9	0.1502	1.2068	0.5315	0.026*	
C10	0.10459 (10)	0.7686 (5)	0.34665 (18)	0.0193 (6)	
H10	0.0930	0.6575	0.2981	0.023*	
C11	0.00282 (10)	0.8103 (5)	0.09670 (18)	0.0180 (6)	
C12	0.00079 (10)	0.8823 (5)	0.01221 (17)	0.0169 (6)	
H12	0.0006	0.7540	−0.0257	0.020*	
C13	0.22077 (10)	0.8447 (5)	0.56670 (18)	0.0205 (6)	

### Atomic displacement parameters (Å^2^)

**Table:**

	*U*^11^	*U*^22^	*U*^33^	*U*^12^	*U*^13^	*U*^23^
Cu1	0.0147 (2)	0.0154 (3)	0.0090 (2)	0.000	0.00500 (19)	0.000
O1	0.0667 (16)	0.0171 (11)	0.0272 (12)	−0.0040 (11)	0.0324 (12)	−0.0003 (9)
O2	0.0219 (11)	0.0249 (12)	0.0250 (12)	0.0040 (9)	0.0022 (9)	0.0016 (10)
O3	0.058 (2)	0.0146 (16)	0.0144 (16)	0.000	0.0181 (16)	0.000
O4	0.0193 (10)	0.0222 (11)	0.0111 (9)	−0.0034 (8)	0.0086 (8)	−0.0021 (9)
N1	0.0373 (16)	0.0429 (18)	0.0212 (15)	−0.0081 (14)	0.0030 (13)	0.0075 (13)
N2	0.0191 (13)	0.0230 (15)	0.0187 (13)	0.0024 (11)	0.0020 (11)	0.0017 (11)
N3	0.0172 (12)	0.0188 (13)	0.0120 (11)	−0.0005 (10)	0.0073 (10)	−0.0011 (10)
C1	0.0261 (17)	0.043 (2)	0.0208 (16)	−0.0033 (15)	0.0005 (14)	−0.0006 (15)
C2	0.0243 (17)	0.035 (2)	0.0302 (19)	−0.0102 (14)	0.0033 (15)	−0.0011 (15)
C3	0.0307 (18)	0.0241 (18)	0.0255 (18)	−0.0030 (14)	0.0068 (15)	0.0037 (14)
C4	0.0262 (17)	0.0343 (19)	0.0206 (16)	−0.0115 (15)	0.0060 (14)	0.0012 (14)
C5	0.0144 (14)	0.0256 (17)	0.0171 (15)	0.0011 (12)	0.0050 (12)	−0.0024 (12)
C6	0.0153 (14)	0.0218 (15)	0.0169 (15)	0.0008 (12)	0.0060 (12)	0.0019 (12)
C7	0.0193 (15)	0.0225 (16)	0.0172 (15)	0.0030 (12)	0.0082 (12)	−0.0008 (13)
C8	0.0159 (14)	0.0199 (16)	0.0214 (16)	0.0017 (12)	0.0080 (13)	−0.0020 (12)
C9	0.0195 (15)	0.0234 (16)	0.0156 (15)	−0.0008 (12)	0.0040 (12)	−0.0076 (12)
C10	0.0223 (15)	0.0201 (16)	0.0158 (15)	−0.0008 (12)	0.0094 (13)	−0.0035 (12)
C11	0.0209 (15)	0.0160 (15)	0.0168 (15)	0.0057 (12)	0.0088 (12)	0.0027 (12)
C12	0.0215 (14)	0.0157 (13)	0.0154 (15)	−0.0008 (12)	0.0103 (12)	−0.0035 (12)
C13	0.0161 (14)	0.0241 (17)	0.0176 (15)	0.0023 (13)	0.0051 (12)	0.0020 (13)

### Geometric parameters (Å, º)

**Table:**

Cu1—O4^i^	1.9485 (17)	C2—C3	1.388 (4)
Cu1—O4	1.9485 (17)	C2—H2	0.9500
Cu1—N3	2.024 (2)	C3—C5	1.379 (4)
Cu1—N3^i^	2.024 (2)	C3—H3	0.9500
Cu1—O3	2.151 (3)	C4—C5	1.380 (4)
O1—C11	1.244 (3)	C4—H4	0.9500
O2—C13	1.222 (3)	C6—C9	1.380 (4)
O3—H3O^i^	0.839 (11)	C6—C8	1.382 (4)
O3—H3O	0.839 (11)	C6—C13	1.500 (4)
O4—C11	1.278 (3)	C7—C9	1.383 (4)
N1—C1	1.330 (4)	C7—H7	0.9500
N1—C4	1.331 (4)	C8—C10	1.378 (4)
N2—C13	1.356 (4)	C8—H8	0.9500
N2—C5	1.423 (3)	C9—H9	0.9500
N2—H2N	0.883 (17)	C10—H10	0.9500
N3—C7	1.335 (3)	C11—C12	1.489 (4)
N3—C10	1.344 (3)	C12—C12^ii^	1.323 (5)
C1—C2	1.374 (4)	C12—H12	0.9500
C1—H1	0.9500		
			
O4^i^—Cu1—O4	176.95 (12)	N1—C4—C5	123.0 (3)
O4^i^—Cu1—N3	88.74 (8)	N1—C4—H4	118.5
O4—Cu1—N3	91.04 (8)	C5—C4—H4	118.5
O4^i^—Cu1—N3^i^	91.04 (8)	C3—C5—C4	118.6 (3)
O4—Cu1—N3^i^	88.74 (8)	C3—C5—N2	119.8 (3)
N3—Cu1—N3^i^	171.71 (13)	C4—C5—N2	121.5 (3)
O4^i^—Cu1—O3	91.52 (6)	C9—C6—C8	118.5 (2)
O4—Cu1—O3	91.52 (6)	C9—C6—C13	122.6 (3)
N3—Cu1—O3	94.15 (6)	C8—C6—C13	118.8 (2)
N3^i^—Cu1—O3	94.15 (6)	N3—C7—C9	122.8 (3)
H3O^i^—O3—Cu1	122.6 (17)	N3—C7—H7	118.6
H3O^i^—O3—H3O	115 (3)	C9—C7—H7	118.6
Cu1—O3—H3O	122.6 (17)	C10—C8—C6	119.4 (3)
C11—O4—Cu1	123.39 (17)	C10—C8—H8	120.3
C1—N1—C4	117.9 (3)	C6—C8—H8	120.3
C13—N2—C5	125.5 (2)	C6—C9—C7	118.9 (3)
C13—N2—H2N	119 (2)	C6—C9—H9	120.5
C5—N2—H2N	115 (2)	C7—C9—H9	120.5
C7—N3—C10	118.1 (2)	N3—C10—C8	122.3 (3)
C7—N3—Cu1	118.25 (18)	N3—C10—H10	118.9
C10—N3—Cu1	123.06 (18)	C8—C10—H10	118.9
N1—C1—C2	123.3 (3)	O1—C11—O4	125.0 (3)
N1—C1—H1	118.4	O1—C11—C12	118.1 (2)
C2—C1—H1	118.4	O4—C11—C12	116.9 (2)
C1—C2—C3	118.4 (3)	C12^ii^—C12—C11	122.7 (3)
C1—C2—H2	120.8	C12^ii^—C12—H12	118.6
C3—C2—H2	120.8	C11—C12—H12	118.6
C5—C3—C2	118.8 (3)	O2—C13—N2	123.7 (3)
C5—C3—H3	120.6	O2—C13—C6	121.1 (3)
C2—C3—H3	120.6	N2—C13—C6	115.2 (2)
			
O4^i^—Cu1—O3—H3O^i^	22 (3)	C13—N2—C5—C4	−36.8 (4)
O4—Cu1—O3—H3O^i^	−158 (3)	C10—N3—C7—C9	−0.6 (4)
N3—Cu1—O3—H3O^i^	−66 (3)	Cu1—N3—C7—C9	170.8 (2)
N3^i^—Cu1—O3—H3O^i^	114 (3)	C9—C6—C8—C10	0.6 (4)
N3—Cu1—O4—C11	121.8 (2)	C13—C6—C8—C10	177.4 (3)
N3^i^—Cu1—O4—C11	−49.9 (2)	C8—C6—C9—C7	0.3 (4)
O3—Cu1—O4—C11	−143.99 (19)	C13—C6—C9—C7	−176.4 (3)
O4^i^—Cu1—N3—C7	−50.8 (2)	N3—C7—C9—C6	−0.3 (4)
O4—Cu1—N3—C7	132.2 (2)	C7—N3—C10—C8	1.5 (4)
O3—Cu1—N3—C7	40.6 (2)	Cu1—N3—C10—C8	−169.4 (2)
O4^i^—Cu1—N3—C10	120.2 (2)	C6—C8—C10—N3	−1.5 (4)
O4—Cu1—N3—C10	−56.8 (2)	Cu1—O4—C11—O1	−24.5 (4)
O3—Cu1—N3—C10	−148.4 (2)	Cu1—O4—C11—C12	153.62 (18)
C4—N1—C1—C2	0.4 (5)	O1—C11—C12—C12^ii^	156.4 (3)
N1—C1—C2—C3	0.1 (5)	O4—C11—C12—C12^ii^	−21.9 (5)
C1—C2—C3—C5	−0.8 (5)	C5—N2—C13—O2	−6.2 (4)
C1—N1—C4—C5	−0.1 (5)	C5—N2—C13—C6	174.0 (2)
C2—C3—C5—C4	1.0 (5)	C9—C6—C13—O2	150.1 (3)
C2—C3—C5—N2	178.0 (3)	C8—C6—C13—O2	−26.5 (4)
N1—C4—C5—C3	−0.6 (5)	C9—C6—C13—N2	−30.1 (4)
N1—C4—C5—N2	−177.6 (3)	C8—C6—C13—N2	153.3 (3)
C13—N2—C5—C3	146.3 (3)		

Symmetry codes: (i) −*x*, *y*, −*z*+1/2; (ii) −*x*, −*y*+2, −*z*.

### Hydrogen-bond geometry (Å, º)

**Table:**

*D*—H···*A*	*D*—H	H···*A*	*D*···*A*	*D*—H···*A*
O3—H3*O*···O1^iii^	0.84 (1)	1.83 (1)	2.660 (2)	169 (3)
N2—H2*N*···O2^iii^	0.88 (2)	2.33 (2)	3.153 (3)	155 (3)
C2—H2···O4^iv^	0.95	2.48	3.360 (4)	153
C7—H7···O1^v^	0.95	2.48	3.430 (4)	178
C9—H9···N1^vi^	0.95	2.39	3.263 (4)	153
C12—H12···O1^vii^	0.95	2.43	3.374 (4)	171

Symmetry codes: (iii) *x*, *y*+1, *z*; (iv) −*x*+1/2, −*y*+5/2, −*z*+1; (v) −*x*, *y*+1, −*z*+1/2; (vi) −*x*+1/2, *y*+1/2, −*z*+3/2; (vii) −*x*, −*y*+1, −*z*.
